# Production of fungal and bacterial growth modulating secondary metabolites is widespread among mycorrhiza-associated streptomycetes

**DOI:** 10.1186/1471-2180-12-164

**Published:** 2012-08-02

**Authors:** Silvia D Schrey, Eric Erkenbrack, Elisabeth Früh, Svenja Fengler, Kerstin Hommel, Nadine Horlacher, Dirk Schulz, Margret Ecke, Andreas Kulik, Hans-Peter Fiedler, Rüdiger Hampp, Mika T Tarkka

**Affiliations:** 1 IMIT-Physiological Ecology of Plants, Auf der Morgenstelle 1, 72076, Tuebingen, Germany; 2 IMIT-Microbiology/Antibiotics, Auf der Morgenstelle 28, 72076, Tuebingen, Germany; 3 Department of Soil Ecology, UFZ-Helmholtz-Centre for Environmental Research, Theodor Lieser Strasse 4, Halle, Germany

## Abstract

**Background:**

Studies on mycorrhiza associated bacteria suggest that bacterial-fungal interactions play important roles during mycorrhiza formation and affect plant health. We surveyed *Streptomyces* Actinobacteria, known as antibiotic producers and antagonists of fungi, from Norway spruce mycorrhizas with predominantly *Piloderma* species as the fungal partner.

**Results:**

Fifteen *Streptomyces* isolates exhibited substantial variation in inhibition of tested mycorrhizal and plant pathogenic fungi (*Amanita muscaria, Fusarium oxysporum*, *Hebeloma cylindrosporum*, *Heterobasidion abietinum, Heterobasidion annosum*, *Laccaria bicolor, Piloderma croceum*). The growth of the mycorrhiza-forming fungus *Laccaria bicolor* was stimulated by some of the streptomycetes, and *Piloderma croceum* was only moderately affected. Bacteria responded to the streptomycetes differently than the fungi. For instance the strain *Streptomyces* sp. AcM11, which inhibited most tested fungi, was less inhibitory to bacteria than other tested streptomycetes. The determined patterns of *Streptomyces*-microbe interactions were associated with distinct patterns of secondary metabolite production. Notably, potentially novel metabolites were produced by strains that were less antagonistic to fungi. Most of the identified metabolites were antibiotics (e.g. cycloheximide, actiphenol) and siderophores (e.g. ferulic acid, desferroxiamines). Plant disease resistance was activated by a single streptomycete strain only.

**Conclusions:**

Mycorrhiza associated streptomycetes appear to have an important role in inhibiting the growth of fungi and bacteria. Additionally, our study indicates that the *Streptomyces* strains, which are not general antagonists of fungi, may produce still un-described metabolites.

## Background

Plant growth is influenced by the presence of bacteria and fungi, and their interactions are particularly common in the rhizospheres of plants with high relative densities of microbes [1]. Pro- and eukaryotic microorganisms compete for simple plant-derived substrates and have thus developed antagonistic strategies. Bacteria have found niches with respect to the utilization of fungal-derived substrates as well, with their nutritional strategies ranging from hyphal exudate consumption to endosymbiosis and mycophagy [2,3]. Current applications related to bacterial-fungal interactions include biocontrol of fungal plant diseases [4] and controlled stimulation of mycorrhizal infection [5]. Better insight into the co-existence mechanisms of soil bacteria and fungi is crucial in order to improve existing applications and to invent new ones.

Abundant in the rhizospheres of plants, the streptomycetes are best known for their capacity to control plant diseases (reviewed by [6,7]). The fact that many streptomycetes are able to produce antifungal compounds indicates that they may be competitors of fungi. Direct inhibition of fungal parasites may lead to plant protection and is often based on antifungal secondary metabolites [8,9]. In parallel to antibiotics, the streptomycetes produce a repertoire of other small molecules, including for instance root growth-inducing auxins [10] and iron acquisition-facilitating siderophores [11].

Ectomycorrhiza formation between filamentous fungi and forest tree roots is crucial to satisfying the nutritional needs of forest trees [12]. The ectomycorrhizas (EM) and the symbiotic fungal mycelia, the mycorrhizosphere, are associated with diverse bacterial communities. Until now, studies on the functional significance of EM associated bacteria have been rare [13-15]. Nevertheless, diverse roles have been implicated for these bacteria, including stimulation of EM formation, improved nutrient acquisition and participation in plant protection (reviewed in [5]).

An important question to be addressed with EM associated bacteria is whether there is a specific selection for particular bacterial strains by mycorrhizas, since this would indicate an established association between the bacteria, the EM fungus, and/or the plant root. Frey-Klett et al. [13] observed such interdependency: the community of fluorescent pseudomonads from EM with the fungus *Laccaria bicolor* was more antagonistic against plant pathogenic fungi than the bulk soil community. This suggested that mycorrhiza formation does select for antifungal compound-producing pseudomonads from the soil. Moreover, these bacteria were not particularly inhibitory to ectomycorrhiza formation with *L. bicolor*, indicating some form of adaptation of this ectomycorrhizal fungus to the *Pseudomonas* community.

Fungus specificity, i.e. selective inhibition or inhibition of one but stimulation of another fungus, is commonly observed in bacterium-fungus co-culture bioassays. Garbaye and Duponnois [14], for instance, observed that bacteria which stimulate growth and mycorrhiza formation by *L. bicolor* may be inhibitory to *Hebeloma cylindrosporum*.To date, the study on metabolites related to fungus specificity of mycorrhiza associated bacteria has focused on one *Streptomyces* isolate. Riedlinger et al. [16] observed that *Streptomyces* sp. AcH 505 stimulated the growth of the mutualist *Amanita muscaria*, while inhibiting the plant parasite *Heterobasidion annosum*[17]. EM formation with *A. muscaria* was stimulated by *Streptomyces* sp. AcH 505, and at the same time Norway spruce roots were protected from *H. annosum* root rot by the same strain [15]. The sole inhibition of *H. annosum* was related to its low level of tolerance to an exudate produced by AcH 505, an antifungal substance WS-5995 B. This indicates that production of antibiotics by mycorrhiza associated bacteria is of central importance in relation to fungus specificity, controlled stimulation of mycorrhizal infection, and plant protection.

There is evidence that inoculation of roots with non-pathogenic bacteria may render plants disease resistant. This phenomenon was studied in detail in the interaction between *Arabidopsis thaliana* and fluorescent pseudomonads and has been termed “priming” [18]. Streptomycetes have also been implicated in the induction of a priming-like state in plants. The inoculation of *Arabidopsis* seedlings with *Streptomyces* sp. EN27 led to suppression of *Fusarium oxysporum* wilt disease in roots and *Erwinia carotovora* soft rot in leaves [19]. Upon pathogen challenge, the endophyte-treated plants demonstrated higher levels of defence gene expression compared with the non-*Streptomyces*-treated controls, indicating a priming-like state in the plant. *Streptomyces* sp. GB 4-2 acted in a similar manner against *Heterobasidion* root and butt rot in Norway spruce seedlings [20]. While the sole inoculation with the plant pathogen led to the lysis of the roots, an anatomical barrier against the root pathogen was formed in the presence of *Streptomyces* GB 4-2. The needles of Norway spruce were also protected from *Botrytis cinerea* gray mold infection, indicating a systemic response.

Here, we report an assessment study of fungal, bacterial, and plant responses to mycorrhiza-associated streptomycetes. Based on our earlier work with mycorrhizosphere streptomycetes [15,20-22], we formulated the following hypotheses: (i) streptomycetes impact fungi and bacteria in a streptomycete strain specific manner, (ii) few strains promote the growth of mycorrhizal fungi, and (iii) induction of plant defence responses is not widespread among streptomycetes.

We restricted our investigations to the genus *Streptomyces*, since it includes well known antagonists of fungi [23], as well as isolates which affect plant resistance against microbial pathogens [15,19,20] and stimulate mycorrhiza formation [22,24]. Since production of multiple secondary metabolites is commonplace in *Streptomyces* species [25] we expected that the mechanisms underlying fungal specificity are related to the specific patterns of secondary metabolite production.

## Results

### *Picea abies* ectomycorrhizas host a community of streptomycetes

Ectomycorrhizas were collected from beneath 10-year-old Norway spruce (*Picea abies*) trees and cleaned from debris under sterile water. White and pale yellow ectomycorrhizal root tips were pooled and the pooled sample was halved in two. Genomic DNA was extracted from the first half and the fungal internal transcribed spacer (ITS) regions were analyzed. Two ectomycorrhizal fungal species were identified from the ectomycorrhizas by blastn comparisons with reference sequence data maintained at NCBI and Unite sequence databases (Additional file 1). These included *Piloderma* sp., which constituted 90%, and *Cortinarius spilomeus*, which constituted 10% of the analyzed sequences (Genbank accessions JF313417-JF313427). Streptomycete cultures were recovered from the second half of the sample. Based on morphological appearance of the sporulating actinomycete isolates on ISP-2 medium, 15 isolates could be distinguished. Partial 16 S rDNA sequencing was used to identify the actinobacterial isolates to the genus level. This placed the isolates in the genus *Streptomyces*. Based on blastn searches with 16 S rDNA reference data from the NCBI database grouped the sequences in seven groups, with 16 S rDNA sequence homology to *S. atratus, S. candidus,, S. hebeiensis, S. drozdowiczii, S. microflavus, S. spiroverticillatus,* and *S. zaomyceticus* (Table 1)*.*

**Table 1:** Picea abies ectomycorrhiza associated streptomycetes

**Strain**	**Closest 16 S rDNA homologue**	**Sequence Identity**	**Genbank accession**
AcM1	*Streptomyces atratus*	99%	JF313428
AcM5	*Streptomyces zaomyceticus*	97%	JF313429
AcM8	*Streptomyces zaomyceticus*	97%	JF313430
AcM9	*Streptomyces microflavus*	98%	JF313431
AcM11	*Streptomyces microflavus*	99%	JF313432
AcM12	*Streptomyces spiroverticillatus*	99%	JF313433
AcM20	*Streptomyces microflavus*	98%	JF313435
AcM25	*Streptomyces spiroverticillatus*	99%	JF313436
AcM29	*Streptomyces hebeiensis*	98%	JF313437
AcM30	*Streptomyces drozdowiczii*	98%	JF313438
AcM31	*Streptomyces drozdowiczii*	98%	JF313439
AcM33	*Streptomyces drozdowiczii*	98%	JF313440
AcM34	*Streptomyces spiroverticillatus*	99%	JF313441
AcM35	*Streptomyces hebeiensis*	98%	JF313442
AcM37	*Streptomyces spiroverticillatus*	99%	JF313443

Partial 16 S rDNA was amplified from pure cultures of bacteria which were isolated from *Picea abies-Piloderma* sp. and *P. abies-Cortinarius spilomeus* ectomycorrhizas. Bacterial isolate number, closest 16 S rDNA homologue of a cultured bacterium, the extent of sequence identity in a region of 580 nucleotides to the closest 16 S rDNA homologue sequence, and Genbank accession of the partial 16 S rDNA fragment are indicated.

### Fungi from the mycorrhizosphere are specific in their responses to the streptomycetes and none of the streptomycete isolates inhibits all fungi

**Figure 1:**
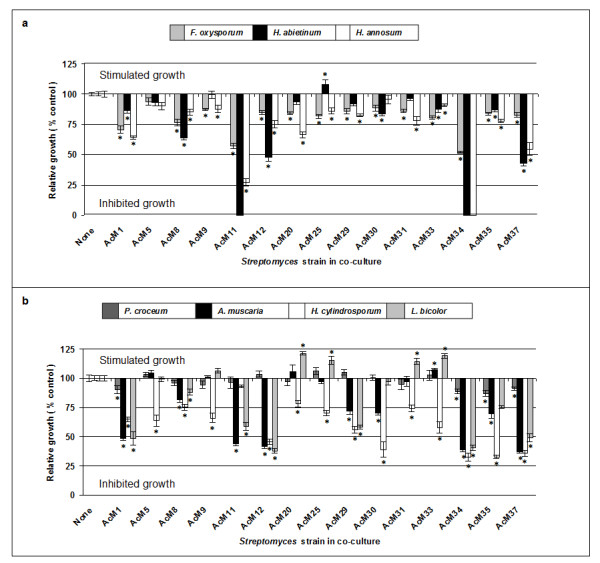
**Influence of streptomycetes on the growth of plant pathogenic and ectomycorrhizal fungi.** The plant pathogenic fungi (**a**) *Fusarium oxysporum, Heterobasidion abietinum* and *Heterobasidion annosum* were cultured for one week, and the mycorrhizal fungi (**b**) *Amanita muscaria, Hebeloma cylindrosporum* and *Laccaria bicolor,* were cultured for eight weeks with Norway spruce ectomycorrhiza associated streptomycete isolates. The extension of fungal mycelium was measured, and related to the treatment without bacteria (None = value 100). Mean and standard error of each experiment with at least 5 replicates are indicated. Signficant difference in mycelial growth in comparison to control without bacterial inoculation, determined by one way analysis of variance (p < 0.05), is indicated by asterisks.

Streptomycete-fungus co-culture bioassays were performed to determine the influence of the bacteria on spruce pathogenic fungi (Figure 1a) and on ectomycorrhizal fungi (Figure 1b). Several antagonists of *Fusarium oxysporum, Heterobasidion abietinum* and *H. annosum* were detected (Figure 1a). Instantly recognizable was the strong suppression of *Heterobasidion* strains by isolates AcM11 and AcM34, associated with significant inhibition of *F. oxysporum*. In general, the two *Heterobasidion* strains responded somewhat differentially to bacterial treatments. While suppression of *H. abietinum* was marked with isolates AcM37 (42% growth rate), AcM12 (47%), and AcM08 (64%), co-cultures of *H. annosum* with the same bacteria led to less inhibition (54%, 75% and 85%, respectively, growth rate compared to the pure culture mycelium). In co-cultures with AcM01 and AcM35, in contrast, mycelial growth of *H. abietinum* was less inhibited than that of *H. annosum.* Growth of *H. abietinum* was promoted by AcM25 while none of the other plant pathogenic fungi showed a positive response to the bacteria.

Qualitative differences were observed between the responses of the tested mycorrhizal fungi towards the streptomycetes (Figure 1b). *Laccaria bicolor* was promoted by four and inhibited by seven bacteria, *Amanita muscaria* and *Piloderma croceum* were inhibited by nine and three strains, respectively, but not promoted. *Hebeloma cylindrosporum* was, in general, inhibited. The bacterial strains AcM1, AcM8, AcM11, AcM34, AcM35 and AcM37 inhibited all symbiotic fungi.

### Strain specific patterns of inhibition in *Streptomyces-Streptomyces* interaction bioassays

**Figure 2:**
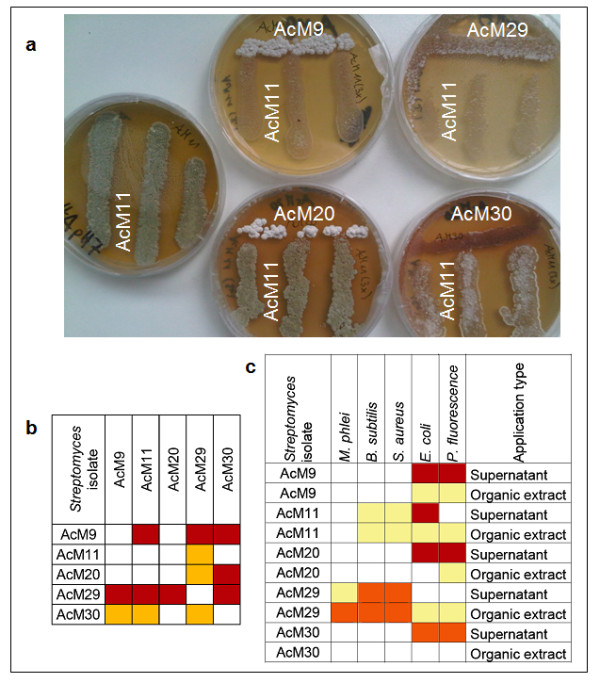
**Bioassay evaluation of the antagonistic activities of five mycorrhiza associated*Streptomyces*isolates against bacteria.** (**a**) Examples of co-culture phenotypes between the mycorrhiza associated *Streptomyces* isolates. AcM11 was confronted with other streptomycetes. (**b**) Degrees of inhibition between five mycorrhiza associated *Streptomyces* isolates. The bacteria were challenged with each other in a Petri dish co-culture bioassay (n = 9). The left hand column equals the singular line while the top row equals the three lines in the Petri dish. Box colours represent the degree of inhibition. No inhibition, white; inhibition of sporulation, yellow; inhibition of growth, red. (**c**) Antibiotic activity of five mycorrhiza associated *Streptomyces* isolates against non-*Streptomyces* bacteria. Gram-positive *Mycobacterium phlei, Bacillus subtilis* and *Staphylococcus aureus*, and Gram-negative *Escherichia coli* and *Pseudomonas fluorescence* were cultivated on agar medium and challenged by either the supernatant or the organic extract of a *Streptomyces* isolate, applied on a filter paper. Boxes represent average zones of inhibition (ZOI) by a given treatment and different colours indicate the degree of inhibition. ZOI = 0-2.5 mm, white; ZOI = 2.6-7.5 mm, light yellow; ZOI = 7.6-12.5 mm, orange; ZOI = 12.6-24 mm, red. Results are based on two separate repetitions with 3 Petri dishes each containing seven filter papers.

In order to assess the interactions between streptomycetes and other bacteria in more detail and to approach the chemical diversity of the streptomycetes, five *Streptomyces* strains were selected for further studies according to their differential impact on fungal growth. These were AcM9, AcM11, AcM20, AcM29 and AcM30. First, co-culture bioassays were used to evaluate how the five *Streptomyces* strains affect each other (Figure 2a, b). AcM29 inhibited all other strains and AcM9 inhibited all except for AcM20. The least inhibitory strain was AcM11, which suppressed sporulation of AcM29.

To mimic the activity of the compound blends produced by *Streptomyces* strains and to compare the inhibition by polar and non-polar compounds we tested culture supernatants and organic culture extract concentrates against Gram-positive and Gram-negative bacteria (Figure 2c). AcM29 inhibited Gram-positive bacteria and other strains suppressed Gram-negative bacteria. Again, the least inhibitory strain was AcM11, which suppressed *Escherichia coli* only. The growth of none of these bacteria was promoted by the streptomycetes. The inhibitory effect of the supernatants of strains AcM9 and AcM20 was distinctly stronger than that of the concentrated organic extract, indicating the involvement of polar substances in antagonism of these strains against bacteria.

### *Streptomyces* strains produce distinct secondary metabolites

**Figure 3:**
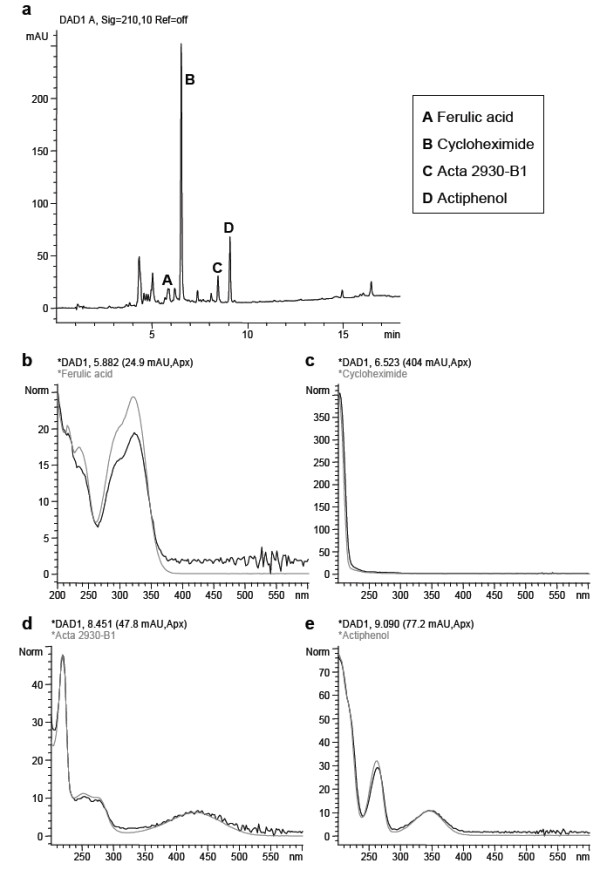
**The strong antagonist of fungi,*Streptomyces*AcM11, produces several antifungal metabolites.** Total ion chromatogram (**a**) and UV/Vis spectra of the peaks A-D (**b**-**e**), extracted from AcM11 oat meal suspension culture. The identities of the metabolites were determined based on their retention times, UV–vis spectra, mass spectrometry, and comparisons to reference compounds.

In order to investigate the secondary metabolite profiles of AcM9, AcM11, AcM20, AcM29 and AcM30, bacterial suspension cultures were grown in two culture media. We found distinct mixtures of secondary metabolites (Table 2). AcM11 produced the antibiotics cycloheximide, actiphenol and Acta 2930 B1 (Figure 3; Additional files 2 and 3). The siderophore ferulic acid was produced by AcM11 and AcM29, and the siderophore desferrioxamine B by AcM29. Other identified metabolites included the tryptophan precursor anthranilic acid and macrolactam antibiotic silvalactam, both produced by AcM30. Most of the metabolites were not identifiable according to the retention time, UV–vis spectrum, and ESI-LC-MS analysis. Apart from the listed metabolites used for mass spectrometry analyses, the *Streptomyces* strains produced further compounds which resulted in the following numbers of peaks: AcM9, five; AcM11, nine; AcM20, eight; AcM29, eleven; AcM30, six. 

**Table 2:** **Chemical diversity of Norway spruce mycorrhiza associated*Streptomyces***

**Strain**	**Medium**	**Substance based on UV–vis**	**Measured [M + H]+**	**Theoretical [M + H]+**	**Confirmed**
**AcM9**	SGG	Unknown	180,1	n. a.	n. a.
**AcM11**	OM	Cycloheximide	282,1	282,169825	Yes
**AcM11**	OM	Actiphenol	276,1	276,123525	Yes
**AcM11**	OM	Acta 2930 B1	1007,5	1008,507825	No
**AcM11**	OM	Ferulic acid	195	195,065735	Yes
**AcM11**	OM	Unknown	292	n. a.	n. a.
**AcM11**	OM	Unknown	407	n. a.	n. a.
**AcM11**	OM	Unknown	387	n. a.	n. a.
**AcM20**	SGG	Unknown	180,1	n. a.	n. a.
**AcM20**	OM	Unknown	298	n. a.	n. a.
**AcM29**	SGG	Desferrioxamine B	561,5	561,691825	Yes
**AcM29**	SGG	Unknown	180	n. a.	n. a.
**AcM29**	SGG	Unknown	340	n. a.	n. a.
**AcM29**	SGG	Unknown	523	n. a.	n. a.
**AcM29**	SGG	Unknown	482	n. a.	n. a.
**AcM29**	OM	Ferulic acid	195,1	195,065735	Yes
**AcM29**	OM	Unknown	298,3	n. a.	n. a.
**AcM29**	OM	Unknown	477,3	n. a.	n. a.
**AcM29**	OM	Unknown	151,1	n. a.	n. a.
**AcM29**	OM	Unknown	217,2	n. a.	n. a.
**AcM30**	SGG	Anthranilic acid	138	138,054825	Yes
**AcM30**	SGG	Silvalactam	637,6	637,427825	Yes

The metabolite spectra of five selected *Streptomyces* strains were investigated. The bacteria were grown on oat meal (OM) and starch-glucose-glycerol (SGG) media. The substances were identified based on their UV–vis spectra and on their molecular mass, determined by ESI-LC-MS. The term “Confirmed” refers to verification of compound identity by comparison with the purified substance. Apart from the listed metabolites the *Streptomyces* strains produced the following numbers of other peaks: AcM9, five; AcM11, nine; AcM20, eight; AcM29, eleven; AcM30, six.

### Varying sensitivity of *Heterobasidion* spp*.* to cycloheximide is reflected in bioassays with the cycloheximide producer *Streptomyces* sp. AcM11

The plant pathogenic fungus *H. abietinum* was more strongly inhibited by AcM11 than *H. annosum* in co-culture. The identification of cycloheximide as an AcM11 produced substance enabled us to assess the tolerance of each fungus to cycloheximide. Cycloheximide concentration in the suspension culture medium was estimated as 10.2 nmol x ml^-1^ (10.2 μM). Based on this finding, a concentration series of cycloheximide was applied. *H. abietinum* was inhibited by 10-fold lower concentrations of cycloheximide than *H. annosum* (Additional file 4). This indicates that the stronger inhibition of *H. abietinum* in co-culture with AcM11 could be related to cycloheximide production. Substance application experiments with the other three identified compounds produced by AcM11, Acta 2930 B1, actiphenol and ferulic acid, did not affect the growth of *H. abietinum* or *H. annosum* (result not shown).

### Inoculation with *Streptomyces* AcM20 leads to increased photosynthetic yield and decreased brassica black spot symptoms in *Arabidopsis thaliana*

**Figure 4:**
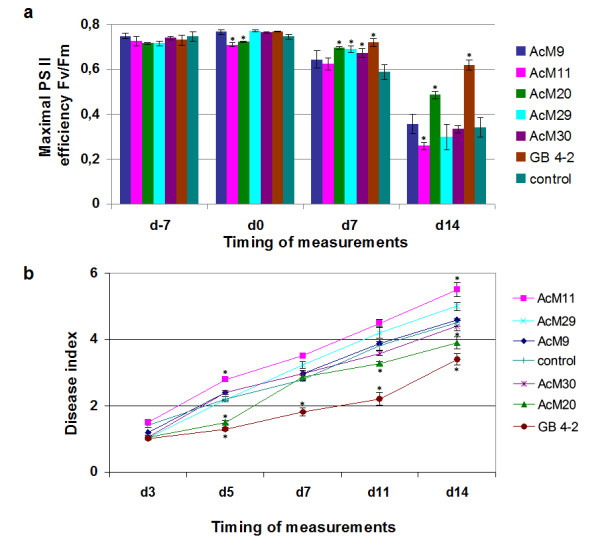
**Treatment with*Streptomyces*sp. AcM20 increases the resistance of*Arabidopsis thaliana*against brassica black spot.***Arabidopsis thaliana* seedlings were preinoculated on roots with streptomycetes or water at d-7 and postinoculated on leaves with *Alternaria brassicicola* at d0*.* Treatment with *Streptomyces* sp. GB 4-2 was included as a positive control, since treatment with GB 4-2 is known to increase the plants’ Fv/Fm value and its disease resistance. In the control treatment no bacteria were inoculated on the roots. (**a**). Plant stress level was estimated according to chlorophyll fluorescence (maximal photon yield of photosystem II), Fv/Fm. At d14, the values with GB 4-2, AcM20 and AcM11 were significantly different from the control treatment (one way analysis of variance, p < 0.05). (**b**). *Alternaria* black spot disease development was determined. At d5, d7, d11 and d14, the values with GB 4-2, at d5, d11 and d14, the values with AcM20 and at d5 and d14, the value of AcM11 were significantly different from the control according to one-way analysis of variance (p < 0.05). Streptomycete strain names are arranged in the top down order of decreasing disease index. Note that a low disease index indicates low amount of fungal infection.

Next we tested the influence of streptomycetes on plant vitality and disease resistance. The photosynthetic yield, Fv/Fm, of *A. thaliana* seedlings was measured as a vitality marker, representing an estimate of the maximum quantum yield of photosystem II in the dark adapted state ( [26]; Figure 4a). The brassica black spot disease index of leaves (Figure 4b) was used as a disease resistance marker. As we have already reported the influence of the *Streptomyces* GB 4-2 on both parameters [20], we included it as a positive control. Similar to *Streptomyces* GB 4-2, we found an increased Fv/Fm value and a decreased disease index after the pre-treatment of the roots with AcM20 (ANOVA, p < 0.05). In contrast, treatment with AcM11 led to decreased Fv/Fm parameter and increased disease index (Figure 4; (ANOVA, p < 0.05). The other tested *Streptomyces* strains did not show any impact on either parameter.

## Discussion

We demonstrated that enrichment isolations of bacteria from *Piloderma-*Norway spruce mycorrhizas encompass chemically diverse streptomycetes. Chemical characterization of the secondary metabolites produced in *Streptomyces* pure cultures revealed structurally diverse compounds, including antifungal and antibacterial compounds as well as siderophores. Our Petri plate bioassay experiments against fungi and bacteria indicated that the observed chemical diversity had an impact on inter-phyletic interactions: the *Streptomyces* strains varied in their antibacterial and antifungal activity. The least inhibited fungus in these bioassays was *Piloderma croceum*, closely related to the mycorrhizal fungus *Piloderma* sp., the fungus which dominated in the Norway spruce mycorrhizal roots used for isolations. This suggests the potential of such a niche-related community for protecting Norway spruce-*Piloderma* mycorrhizas from fungal and bacterial parasites without incurring harm to the host fungus.

### The production of secondary metabolites by mycorrhiza associated streptomycetes

After many years of intensive screening of actinomycetes, the frequency of discovering structurally new compounds is apparently decreasing [27]. Since the current strategies for addressing the urgent need for new antibiotics are not efficient enough, another approach might be to examine new niches, or sources, for microbial resources that produce novel compounds [28]. To search for compounds that affect fungal growth we performed HPLC analyses coupled with UV/Vis detection and mass spectrometry with five selected mycorrhiza-associated streptomycetes, possessing different activities in *Streptomyces*-fungus bioassays. Typically, only a limited number of metabolites are produced in synthetic media [27], and to promote production of diverse metabolites two different culture media were employed. The five strains produced diffusible secondary metabolites, of which only seven could be identified using the HPLC-UV–vis database containing 960 reference compounds [29], NIST database, and MS analyses. The identified metabolites included antifungal and antimicrobial substances as well as siderophores. The fungal inhibitory strain *Streptomyces* AcM11 produced the most characterized metabolites, the antibiotics Acta 2930 B1, actiphenol, cycloheximide and the siderophore ferulic acid. This indicates that function based screening, e.g. selection of isolates that are highly inhibitory towards fungi for biocontrol applications, may create a bias towards strains producing known compounds. Based on spectral measurements and MS analyses, a total of twenty one compounds were produced by the five isolates, suggesting an abundance of yet unreported, putatively bioactive compounds. Nevertheless, at least 7000 secondary metabolites have been discovered from streptomycetes [27], and the genome sequences of *Streptomyces* spp. commonly contain 20-30 gene clusters for secondary metabolite synthesis, of which approximately 30% may encode biochemical pathways for antibiotics production [30]. Thus, to conclusively determine the novelty of such substances both structural and chemical elucidation as well as the use of comprehensive substance databases is indispensable.

### The distinct responses of fungi and bacteria to five selected streptomycete isolates

Five AcM *Streptomyces* isolates were selected for secondary metabolite analyses to test our hypothesis that variation in secondary metabolite production is mirrored in the variation of the observed dual culture phenotype. Thus, general inhibitors of fungi and/or bacteria, selective inhibitors, and a selective fungal growth-promoting strain were chosen. HPLC analyses revealed great differences in substance production. For example, strains 29 and 30 exhibited comparable impacts on fungal growth, yet they differed greatly in the numbers of detected products (10 vs. 2). The strain producing the most unreported metabolites, AcM29, was characterized by a complex *Streptomyces*-fungus interaction spectrum. AcM29 had a negative impact on *A. muscaria, H. cylindrosporum* and *L. bicolor* but did not inhibit plant pathogenic fungi. Streptomycetes and other tested Gram-positive bacteria were inhibited by AcM29, while Gram-negative bacteria were only slightly influenced. This suggests that in search for *Streptomyces* strains producing putatively novel compounds, a preliminary screen should not only target fungi and Gram-negative bacteria, but also the streptomycetes.

*Heterobasidion* infects roots in particular by growing over root to root contacts [31], and the roots of their host trees are predominatly mycorrhizal [12]. Cycloheximide producing streptomycetes on the mycorrhizal roots could thus potentially affect root rot development. We observed that the addition of 1 nmol cycloheximide to the culture medium mimicked the impact of cycloheximide producer AcM11 to *Heterobasidion* species. Neither of the other compounds produced by AcM11 (antibiotic Acta 2930 B1, actiphenol and ferulic acid) affected the growth of *H. abietinum* or *H. annosum,* indicating that cycloheximide is responsible for the observed growth inhibition by AcM11. The role of cycloheximide in the inhibition of *Heterobasidion* species is supported by our study with another cycloheximide producing streptomycete, *Streptomyces* sp. A230 from South Brazilian soil. Whereas *H. abietinum* is killed by A230, *H. annosum* still retains 30% of its growth rate. Interestingly, A230 not only produces cycloheximide, but also actiphenol, a combination also observed in AcM11 (S.D.S, N.H., A. Zander and L. Braun, unpublished). *H. abietinum* and *H. annosum* have been reported to be physiologically and taxonomically distinct species [31]. The data of Lehr et al. [21] indicate that the two species also respond differently to cycloheximide: the levels of gene expression by *H. abietinum* and *H. annosum* are highly distinct upon cycloheximide application. Long-term screening of streptomycetes shows that approximately 10% of *Streptomyces* isolated from soil produce cycloheximide (H.-P. Fiedler, unpublished). It would thus be expected that most fungi have developed resistance or at least tolerance against the antibiotic, since they supposedly regularly encounter cycloheximide producers in the rhizosphere. *P. croceum* and *H. cylindrosporum* were not inhibited by the cycloheximide producer AcM11 (this study) and we recently obtained yeasts during selective isolation of streptomycetes from mushrooms on cycloheximide containing culture media [32], which supports the role of cycloheximide in bacterium-fungus interactions. In addition to a specific activity of a single compound, synergistic effects of complex mixtures of substances exuded by a *Streptomyces* bacterium are likely to occur (reviewed in [33]). For instance, *S. clavuligerus* produces beta-lactamase inhibitors, beta-lactams and cephalosporin analoges that inhibit beta-lactam resistant bacteria only in combination [34].

### The *streptomyces* community includes fungal growth inhibiting and -promoting members

Elo et al. [35] observed that one-third of the *Streptomyces* bacteria from the humus layer of Norway spruce stands possessed antifungal properties on plant pathogenic fungi, and none of the strains promoted the growth of the pathogenic fungi. We obtained similar results with mycorrhiza associated *Streptomyces* bacteria. As stated in our first hypothesis, the impacts of mycorrhiza-derived streptomycetes on fungi and bacteria were *Streptomyces* strain-specific. None of the fifteen AcM isolates inhibited all fungi; four of the strains inhibited some fungi and stimulated the mycorrhizal fungus *Laccaria bicolor*. Dramatic effects were seen only in connection with the plant pathogenic genus *Heterobasidion*, as AcM11 and AcM34 completely blocked the growth of *H. abietinum*.

The occurrence of beneficial interactions between the streptomycetes and the mycorrhizal fungus *Laccaria bicolor* indicate that the presence of potentially interesting positive *Streptomyces*-fungus interactions should not be neglected. Richter et al. [36] used red pine roots for actinomycete isolations, and they observed similar *in vitro* effects on ectomycorrhizal fungi as we did in our analysis. Most actinomycete isolates exerted effects on fungal growth, inhibiting some while stimulating other fungi. Our previous analyses indicate that streptomycetes may produce small molecules that act as fungal growth stimulators. Auxofuran, the compound released by the “Mycorrhization Helper Bacterium” *Streptomyces* AcH 505, promotes the growth of fly agaric [16]. Such growth-promoting *Streptomyces* substances deserve further attention, as does the analyses of the influence of such substances on fungal metabolism and mycorrhiza formation. In nature, an important factor relating to the production of such small molecules is organismic interactions. For instance, higher levels of auxofuran are produced by AcH 505 in dual culture with the fungus *Amanita muscaria*, while the production of the antibiotics WS-5995 B and WS-5995 C, potent inhibitors of fungi, is attenuated [16]. We observed that the *in vivo* interactions between mushroom-associated yeasts are distinctly different in dual and tripartite cultures [32], suggesting that increasing complexity of communities complicates the prediction of the outcomes of interactions and production levels of bioactive substances. Thus, even though several reports indicate a correlation between *in vitro* growth stimulation and mycorrhiza formation [22,37] and *in vitro* growth inhibition and biocontrol [38], the value of tripartite culture systems including the host plant, and a natural substrate, is clear [5,39].

### Plant disease resistance is stimulated by a single *Streptomyces* strain only

Only a single *Streptomyces* strain isolated from the mycorrhizas, AcM20, stimulated plant photosynthetic yield and plant disease resistance against *Alternaria* black spot. Non-pathogenic rhizobacteria, including streptomycetes (reviewed in [7]), have been shown to induce resistance in plants both locally and in distal tissues [19]. However, in comparison to *Streptomyces* GB 4-2, the Norway spruce mycorrhizosphere isolate with positive influence on not only the plants’ disease resistance but also on its photosynthetic yield [20], the response of *Arabidopsis thaliana* to AcM20 was moderate. Plant growth promotion and enhancement of photosynthetic capacity is not a general feature among mycorrhiza-associated streptomycetes. This assumption is supported by the fact that the tested AcM strains, in general, did not affect plant growth. Even the cycloheximide producer AcM11 had only a subtle negative effect on *A. thaliana*, expressed as lower photosynthetic yield and increased black spot disease index.

## Conclusions

*Streptomyces* community from mycorrhizal roots may impact the growth of spruce-associated micro-organisms in a strain specific manner. Differential growth-inhibition was related to the metabolite patterns of each strain, indicating that we have found a novel and a potentially interesting niche for small molecule discovery. We suggest that the combination of antifungals produced by the *Streptomyces* strains from *Piloderma* mycorrhizas provides a broad spectrum of antifungal activity that protects the mycorrhizal roots from fungal parasites, and selects against mycorrhizal fungal competitors.

## Methods

### Isolation of actinomycetes from Norway spruce mycorrhizas

Ectomycorrhizas were collected from beneath 10-year-old Norway spruce (*Picea abies*) trees in a forest stand dominated by Scots pine (*Pinus sylvestris*) in Haigerloch, south-west Germany. Mycorrhizal rootlets from the approx. 5 cm thick organic litter layer were excised, transported on ice to the laboratory, pooled, and subsequently immersed in water to remove debris surrounding the hyphal mantle. After washing 10 times with sterile destilled water, the ectomycorrhizas were sorted and white and pale yellow mycorrhizal root tips were pooled for further study. The mycorrhizal sample was used for both bacterial isolation and the analysis of fungal populations in the mantle. First half of the pooled sample of ectomycorrhizas (0.5 g) was used for DNA extraction according to Doyle and Doyle [40] and sequences of fungal internal transcribed spacer regions were obtained from the ectomycorrhizas with ITS1 and ITS4 primers [41]. The PCR products were cloned and sequenced in two directions at GeneCust (Evry, France) and compared by blastn to sequences at NCBI ( http://www.ncbi.nlm.nih.gov/genbank) and at Unite ( http://unite.ut.ee; [42]) sequence databases.

Second half of the ectomycorrhizas (0.5 g) was used for the isolation of streptomycetes. The mycorrhizal sample was added to 50 ml of HNC medium ( [43]; 6% yeast extract, 0.05% SDS, 0.05% CaCl_2_ pH 7.0) and incubated at 42°C with shaking for 30 min. The suspension was filtered through a fine glass mesh, and a dilution series was subsequently prepared. The filtered suspensions were plated onto ISP-2 agar [44], which contained 5 gL^-1^ cycloheximide, 2 gL^-1^ nalidixic acid, and 5 gL^-1^ nystatin. After 8 d at 27°C fifteen different actinomycete isolates could be distinguished according to their morphological appearance [45], and these were maintained on ISP2 agar. For 16 S rDNA gene sequencing, genomic DNA was extracted from a loopful (a few μl) of bacterial spores by GenElute bacterial genomic DNA extraction kit (Sigma, Schnelldorf, Germany). Partial 16 S rDNA sequence was amplified with the primers 27f (5-AGAGTTTGATCMTGGCTCAG-3) and 765r (5-CTGTTTGCTCCCCACGCTTTC-3) as described in Coombs and Franco [46]. The DNA sequences were compared to NCBI’s nr database and to Greengenes database ( http://greengenes.lbl.gov) by blastn to find the closest homologue for each 16 S rDNA gene fragment from taxonomically characterized homologues. *Streptomyces* sp. GB 4-2, isolated from Schönbuch forest near Tübingen, south-west Germany, was provided by Karl Poralla.

### Fungal isolates, bacterium-fungus co-cultures

The phytopathogenic fungi, *Heterobasidion abietinum* 331 from Klein Kotterbachtal, Austria, *H. annosum* 005 from Kirkkonummi, Finland, obtained from K. Korhonen, and *Fusarium oxysporum* from Schönbuch forest near Tübingen, Germany, obtained from A. Honold, were maintained on 1.5% malt agar. The symbiotic fungi, *Amanita muscaria* strain 404, isolated from fruiting body collected from the Schönbuch forest near Tübingen, Germany, *Hebeloma cylindrosporum* strain H1-H7 [47], and *Laccaria bicolor* strain S238 N [48] were cultivated in the dark at 20 °C on MMN agar [49] with 10 gL^-1^ glucose.

The co-culture system was similar to that utilized by Maier et al. [17], but with some minor alterations. Actinomycetes were spread on MMN medium [49] so as to form a line directly in the middle of the dish, essentially dividing it in two, and were grown at 27°C for 4 days (until sporulation started). Utilizing the wide end of a Pasteur pipette to control for diameter, two plugs of the fungal inoculum were then placed inside the Petri dishes on opposite ends of the plates. Inoculi were allowed to grow for 1 week (fast growing *Heterobasidion* strains and *F. oxysporum*), for 4 weeks (*H. cylindrosporum*) or for 6 weeks (*A. muscaria, L. bicolor* and *P. croceum*). Thereafter the extension of fungal mycelium was recorded from the fungal inoculum to the edge of the colony.

### Confrontation of mycorrhiza-derived *Streptomyces* strains with each other

The influence of five streptomycetes upon each other was tested pair-wise in a bioassay. *Streptomyces* suspension cultures were grown three days in ISP-2 medium. From the tester strain, 40 μl of this suspension culture was applied on the lower part of an agar filled Petri dish, forming a line. After the sporulation of the tester strain begun, 3 parallel lines of the receiver strain were applied perpendicularly to the tester line. For each *Streptomyces* pair, three tester and nine receiver lines were applied. The impact of the tester strain on the formation of receiver strain’s substrate mycelium and sporulation was recorded at the time point of the onset of sporulation in the control cultures.

### Impact of *Streptomyces* culture filtrates and culture extracts on non-streptomycetous bacteria

Pure culture filtrates and organic extracts of streptomycetes were tested against bacteria. *Streptomyces* suspension cultures were grown three days in ISP-2 medium. To obtain pure culture filtrate, the cells were centrifuged (3800 rpm, 10 min), and the supernatants were filtered (0.45 μm). Organic extracts were prepared from the pure culture filtrates, which were adjusted to pH 5.0 and extracted 1:1 (vol/vol) with ethyl acetate. The organic phase was concentrated to dryness using a vacuum evaporator and re-dissolved in 1/10 of the original volume in ethanol.

Gram-positive bacteria (*Bacillus subtilis* DSM 10, *Staphylococcus aureus* DSM 20231, *Mycobacterium phlei* DSM 750) and Gram-negative bacteria (*Escherichia coli* K12 (W1130), *Pseudomonas fluorescens* DSM 50090) were tested. *Bacillus subtilis* DSM 10 was initially cultured in DSMZ 1 medium at 37°C and tested on DSMZ 1 and MM 1 agar media. *Staphylococcus aureus* DSM 20231 was initially cultured in KM 1 medium at 37°C and tested on KM 1 agar medium. *Mycobacterium phlei* DSM 750 was initially cultured in KM 1 medium at 27°C and tested on KM 1 agar medium. *Escherichia coli* K12 (W1130) was initially cultured in KM 1 medium at 37°C and tested on KM 1 and MM 1 agar media. *Pseudomonas fluorescens* DSM 50090 was initially cultured in KM 1 medium at 27°C and tested on KM 1 and MM 1 agar media. KM 1 medium consisted of 8 g Difco nutrient broth, 5 g NaCl, 20 g agar per 1 liter of de-ionized water. The pH was adjusted to pH 7.2 prior to sterilization. KM 5 medium consisted of 4 g yeast extract, 10 g malt extract, 4 g glucose, 20 g agar per liter un-distilled water. The pH was adjusted to pH 5.5 prior to sterilization. DSMZ1-medium consisted of 5 g Bacto peptone, 3 g malt extract, 10 mg MnSO_4_ x H_2_O and 20 g agar per liter of un-distilled water. The pH was adjusted to 5.5 prior to sterilization. MM1 medium [50] consisted of 5 g glucose, 0,5 g tri-sodium-citrate x 2 H_2_O, 3 g KH_2_PO_4_, 7 g K_2_HPO_4_, 0.1 g MgSO_4_ x 7 H_2_O, 1 g (NH_4_)2SO_4_ and 15 g Bacto agar.

The bacteria were cultivated for a period of 24 h in 100 ml in respective liquid media in 500 ml Erlenmeyer flasks with one baffle at 27°C or 37°C on a rotary shaker at120 rpm. The cultures were centrifuged, re-suspended in saline, and set to achieve an optical density of 1.3 at a wavelength of 546 nm. In the case of minimal medium (MM1), cultures were washed one time with saline to get rid of complex media used for inoculation. Two hundred ml of complex medium (DSMZ 1, KM 1, and KM 5) containing agar were inoculated with 2 ml of this defined suspension of organisms (OD = 1.3). Ten ml of inoculated agar were poured into each Petri dish. *Streptomyces* pure culture filtrate (10 μl) or organic extract (10 μl) was applied on paper discs (diameter: 6 mm) and air dried. The paper discs were then placed on the previously prepared agar media. After 24 h, microbial growth inhibition was recorded by measuring the diameter of the inhibition zone.

### Fermentation of streptomycetes for the analysis of secondary metabolites

The strains AcM9, AcM11, AcM20, AcM29 and AcM30 were cultivated in 100 ml ISP-2-medium at 120 rpm and 27 °C for 3 days. Of these cultures, four ml were used to inoculate 100 ml SGG, OM and MMN medium in 500 ml-Erlenmeyer flasks with one baffle. SGG-medium consisted of 10 g soluble starch, 10 g glucose, 10 g glycerol, 2.5 g cornsteep powder (Marcor, Hartge Ingredients, Hamburg), 5 g Bacto peptone (Difco), 2 g yeast extract (Ohly Kat, Deutsche Hefewerke, Hamburg), 1 g NaCl and 3 g CaCO_3_ per liter of tap water. The pH was adjusted to pH 7.3 prior to sterilization. OM medium consisted of 20 g oat meal (Holo Hafergold, Neuform, Zarrentin) and 5 ml of the following micronutrient solution: 3 g CaCl_2_x2 H_2_O, 1 g iron-III-citrat, 200 mg MnSO_4_ x 1 H_2_O, 100 mg ZnCl_2_, 25 mg CuSO_4_ x 5H_2_O, 20 mg Na_2_B_4_O_7_ x 10 H_2_O, 4 mg CoCl_2_ x 6H_2_O, and 10 mg Na_2_MoO_4_ x 2 H_2_O per liter of deionized water. The pH was adjusted to pH 7.3 prior to sterilization. Modified MMN medium was prepared according to Molina and Palmer [49]. Fermentations were carried out on a rotary shaker at 120 rpm and 27°C. After 2, 4 and 6 days (24, 48 and 72 hours) 10 ml of bacterial culture were centrifuged (3800 rpm, 10 min) and bacterial biomass was determined (volume percent). The culture filtrate - separated from the bacterial mycelium by centrifugation - was used for further analyses of secreted bacterial metabolites.

### Extraction and HPLC-UV-visible spectral analysis of *Streptomyces* secondary metabolites

Culture filtrates (5 ml) of AcM 9, AcM11, AcM20, AcM29 and AcM30 were adjusted to pH 5 and extracted with 5 ml ethyl acetate for 30 min under shaking conditions. The organic extracts were concentrated to dryness using vacuum evaporator and resuspended in 0.5 ml of methanol. The 10-fold concentrated extracts were centrifuged (3 min, 13 000 rpm) and 5 μl of each sample was subjected to HPLC on a 5 μm Nucleosil C18-column (Maisch, Ammerbuch, Germany, 125 mm x 3 mm, fitted with a guard-column: 20 mm x 3 mm) with 0.1% -o-phosphoric acid as solvent A and acetonitrile as solvent B at a linear gradient (from 4.5 to 100% B in 15 min and a 3-min hold at 100% B) at a flow rate of 0.85 ml/min. The chromatographic system consisted of a 1090 M liquid chromatograph (Hewlett Packard, Waldbronn, Germany) equipped with a diode array detector and a Kayak XM 600 ChemStation (Agilent Technologies, Waldbronn, Germany). Multiple wavelengths monitoring was performed at 210, 230, 260, 280, 310, 360, 435 and 500 nm and UV-visible spectra were measured from 200 to 600 nm.

### HPLC-ESI-MS analysis of *Streptomyces* secondary metabolites

HPLC-DAD-ESI-MS analysis was carried out with an Agilent 1200 HPLC series equipped with a binary HPLC pump, autosampler and diode array detector, and an Agilent LC/MSD Ultra Trap System XCT 6330 (Agilent, Waldbronn, Germany). The Samples (2.5 μL) were separated on a 3 μm Nucleosil C18-column (Maisch, Ammerbuch, Germany, 100 mm x 2 mm with a precolumn 10 mm x 2 mm) and separated by linear gradient elution from 10% eluent B to 100% eluent B in 15 minutes (0.1% formic acid as eluent A, 0.06% formic acid in acetonitrile as eluent B) at a flow rate of 400 μl/min. Wavelength monitoring was performed at 230 nm, 260 nm, 280 nm, 360 nm and 435 nm. MS Instrument settings were as follows: Ionization: ESI (positive and negative, alternating); Mode: Ultra Scan; Capillary voltage: 3.5 kV; Temperature: 350°C; Tuning mass: m/z 400. The production levels of the following metabolites were quantified based on the comparison of their peak area with that obtained by HPLC analysis of known amount of pure substance: Acta 2930 B1, actiphenol, cycloheximide, ferulic acid.

### Inoculation of *Arabidopsis thaliana* with streptomycetes and with *Alternaria brassicicola*, chlorophyll fluorescence and disease index measurements

Sterile *Arabidopsis thaliana* Col-0 seeds were placed on half strength MS [51] medium containing 1% glucose and 0.8% agar for germination. After 7 days, seedlings were transferred to ½ MS with 2% agar. To grow seedlings in an upright position with leaves free from contact with the agar surface, the top third of solid medium was removed from the Petri dish. Seedlings were placed with roots on the agar and leaves in the airspace. Petri dishes were then stored in a vertical position to allow root growth on the agar surface. Plants were cultivated at 22°C, 200μE/m2s with a light/dark cycle of 8/16 h.

After 7 days, roots were inoculated with AcM 9, AcM11, AcM29, AcM29, AcM30 and positive control *Streptomyces* GB 4-2 [20]. Bacterial cultures grown in ISP-2 medium for 4 to 5 days were separated from growth medium by centrifugation, washed three times in sterile water and diluted to an OD of 0.3. Fourteen μl were applied to each root. Control plants (no bacterial inoculation) received 14 μl of sterile water. This time point was referred to as “d-7” and was the first time point of measurement of maximal photosystem II efficiency (Fv/Fm), which was measured using Imaging-PAM fluorometer (Walz, Effeltrich, Germany) in the following manner: seedlings were subjected to a saturated light impulse of 3000 μE/m^2^s and 0.7 sec duration to establish maximal fluorescence and basic fluorescence, from which maximal Fv/Fm was calculated. Results were based on two values of 10 plants per each time point. Each treatment contained in total 30 plants in three independent repetitions. Standard deviation was calculated based on mean values of those repetitions. Seven days after bacterial inoculation of roots (referred to as “d0”), 2 to 3 leaves of each seedling were infected with 1 μl each of a 5x10^5^ spores/ml suspension of *Alternaria brassicicola* (kindly donated by Birgit Kemmerling, ZMBP, University of Tuebingen).

Disease index was determined regularly from day 3 post *Alternaria brassicicola* infection (d3) based on Epple et al. [52]. The spread of fungal infection on each leaf was assessed at d3, d5, d7, d11, and d14 post *Alternaria brassicicola* inoculation, and quantified in classes 1 to 6: class 1: no infection, class 2: infection restricted to site of inoculation, class 3: symmetric spread of infection around inoculation site, class 4: asymmetric spread of infection around inoculation site, class 5: beginning sporulation of pathogen, and class 6: >50% of leave surface infected. Disease index (DI) was calculated as DI = ∑ *i* x *l*/*n* where *i* is infection class, *l* number of leaves in the respective class and *n* is total number of infected leaves. Results were calculated as mean values of three independent repetitions each containing 20 infected leaves of 10 plants per treatment. Standard deviations were calculated from mean values of independent repetitions.

## Abbreviations

ESI: Electrospray ionization; HPLC: High-performance liquid chromatography; MS: Mass spectrometry; PCR: Polymerase chain reaction; UV–vis: Ultraviolet–visible.

## Competing interests

The authors declare to have no competing interests.

## Authors’ contributions

EE carried out the bacterial isolation and identification, and performed part of fungus-bacterium co-cultures. EF, NH, AK, KH, ME and DS carried out the chemical isolations, applications on microbes, and substance identifications. SF carried out the plant culture experiments. MT and SDS conceived and designed the study, RH, NH and HPF participated in its coordination. MT and SDS prepared the manuscript. All authors read and approved the final manuscript.

## Supplementary Material

Additional file 1 **Analysis of ribosomal DNA sequences from *Picea abies*ectomycorrhiza.** One hundred ectomycorrhizal root tips were pooled and used for the amplification of internal transcribed spacer 1, 5.8 S ribosomal RNA gene and internal transcribed spacer 2. Clone number, closest partial rDNA homologue and Genebank accession are indicated. Click here for file

Additional file 2 **Analysis of metabolites from *Streptomyces* sp. AcM11 Extracts were gained and analyzed as described in Methods.** Total ion chromatograms at ESI-MS positive (a) and negative (b) modes, and UV–vis spectrum at 230-600 nm (c) of organic extracts of *Streptomyces* sp. AcM11 suspension culture. The peaks I, II, III and IV are marked. The averaged masses of the ions within peaks I, II III, and IV are presented in ESI-MS positive (d, f, h, j) and negative (e, g, i, k) modes. The by MS and by comparisons to reference substance identified compounds are indicated by asterisks. Peak I was identified as ferulic acid (MW = 194.06), peak II as cycloheximide (MW = 281.16), peak III as actiphenol (MW = 275.12), and peak IV as a derivative of Acta 2930-B1 (m/z = 1030.5 at [MS + H] + and m/z = 1006.5 at [MS-H]-). Click here for file

Additional file 3 ***Streptomyces* sp. AcM11 produces a derivative of Acta 2930-B1 Comparisons between the chromatogram and the averaged masses of the ions from Acta 2930-B1 pure substance and from peak IV of *Streptomyces* AcM11 extract, prepared as described in Methods.** (a) The chromatogram of Acta 2930-B1 pure substance (blue) and the *Streptomyces* AcM11 extract (red). Average masses of Acta 2930-B1 pure substance and the *Streptomyces* AcM11 extract are in ESI-MS positive (b, d) and negative (c, e) modes. Note that the dominant masses in peak IV deviate one m/z unit from the respective values of the Acta 2930-B1 pure substance. Click here for file

Additional file 4 ***Heterobasidion abietinum *is more sensitive to the cycloheximide producer, *Streptomyces *AcM11, and to cycloheximide than *H. annosum.*** Antifungal influence of AcM11 and cycloheximide was tested in a Petri dish bioassay test against *H. abietinum* 331 and *H. annosum* 005. (a, d) Influence of AcM11 on the growth of the fungus. AcM11 was applied on agar medium and the fungus was inoculated. The front of the fungal colony was circled by pencil. (b, e) Influence of cycloheximide on fungal growth. Methanol or in methanol dissolved cycloheximide was applied by filter paper on the top of the agar medium. Note that *H. abietinum* growth under the influence of 4 nmol cycloheximide is comparable to *H. annosum* growth with 50 nmol cycloheximide. The front of the fungal colony was circled by pencil. (c, f) Influence of cycloheximide on fungal growth on fungal growth. Extension of fungal mycelium was measured after one week of growth on cycloheximide containing medium (n = 9). Cycloheximide concentration range in the bioassay is based on the observed production level in the AcM11 suspension culture, which was 10.2 nmol x ml^-1^. Note the lower levels of cycloheximide applications to *H. abietinum* than to *H. annosum*. Click here for file
