# Supplementary material for: Production of fungal and bacterial growth modulating secondary metabolites is widespread among mycorrhiza-associated streptomycetes
Source: BMC Microbiol. 2012 Aug 2;12:164. doi: 10.1186/1471-2180-12-164 (PMC3487804; doi:10.1186/1471-2180-12-164)
Supplement: Additional file 1 — Analysis of ribosomal DNA sequences from Picea abiesectomycorrhiza. One hundred ectomycorrhizal root tips were pooled and used for the amplification of internal transcribed spacer 1, 5.8 S ribosomal RNA gene and internal transcribed spacer 2. Clone number, closest partial rDNA homologue and Genebank accession are indicated. [file 1471-2180-12-164-S1.doc]

_____________________________________

**rDNA Genbank**

**Clone homologue accession**

_____________________________________

F1 *Piloderma* sp. JF313417

F2 *Piloderma* sp. JF313418

F3 *Piloderma* sp. JF313419

F4 *Piloderma* sp. JF313420

F5 *Piloderma* sp. JF313421

F6 *Cortinarius spilomeus* JF313422

F7 *Piloderma* sp. JF313423

F8 *Cortinarius spilomeus* JF313424

F9 *Piloderma* sp. JF313425

F10 *Piloderma* sp. JF313426

F11 *Piloderma* sp. JF313427

_____________________________________

**Additional File 1 Analysis of ribosomal DNA sequences from *Picea abies* ectomycorrhiza*.*** One hundred ectomycorrhizal root tips were pooled and used for the amplification of internal transcribed spacer 1, 5.8S ribosomal RNA gene and internal transcribed spacer 2. Clone number, closest partial rDNA homologue and Genebank accession are indicated.
