# Supplementary material for: Production of fungal and bacterial growth modulating secondary metabolites is widespread among mycorrhiza-associated streptomycetes
Source: BMC Microbiol. 2012 Aug 2;12:164. doi: 10.1186/1471-2180-12-164 (PMC3487804; doi:10.1186/1471-2180-12-164)
Supplement: Additional file 2 — Analysis of metabolites from Streptomyces sp. AcM11 Extracts were gained and analyzed as described in Methods. Total ion chromatograms at ESI-MS positive (a) and negative (b) modes, and UV–vis spectrum at 230-600 nm (c) of organic extracts of Streptomyces sp. AcM11 suspension culture. The peaks I, II, III and IV are marked. The averaged masses of the ions within peaks I, II III, and IV are presented in ESI-MS positive (d, f, h, j) and negative (e, g, i, k) modes. The by MS and by comparisons to reference substance identified compounds are indicated by asterisks. Peak I was identified as ferulic acid (MW = 194.06), peak II as cycloheximide (MW = 281.16), peak III as actiphenol (MW = 275.12), and peak IV as a derivative of Acta 2930-B1 (m/z = 1030.5 at [MS + H] + and m/z = 1006.5 at [MS-H]-). [file 1471-2180-12-164-S2.pdf]

Additional File 2

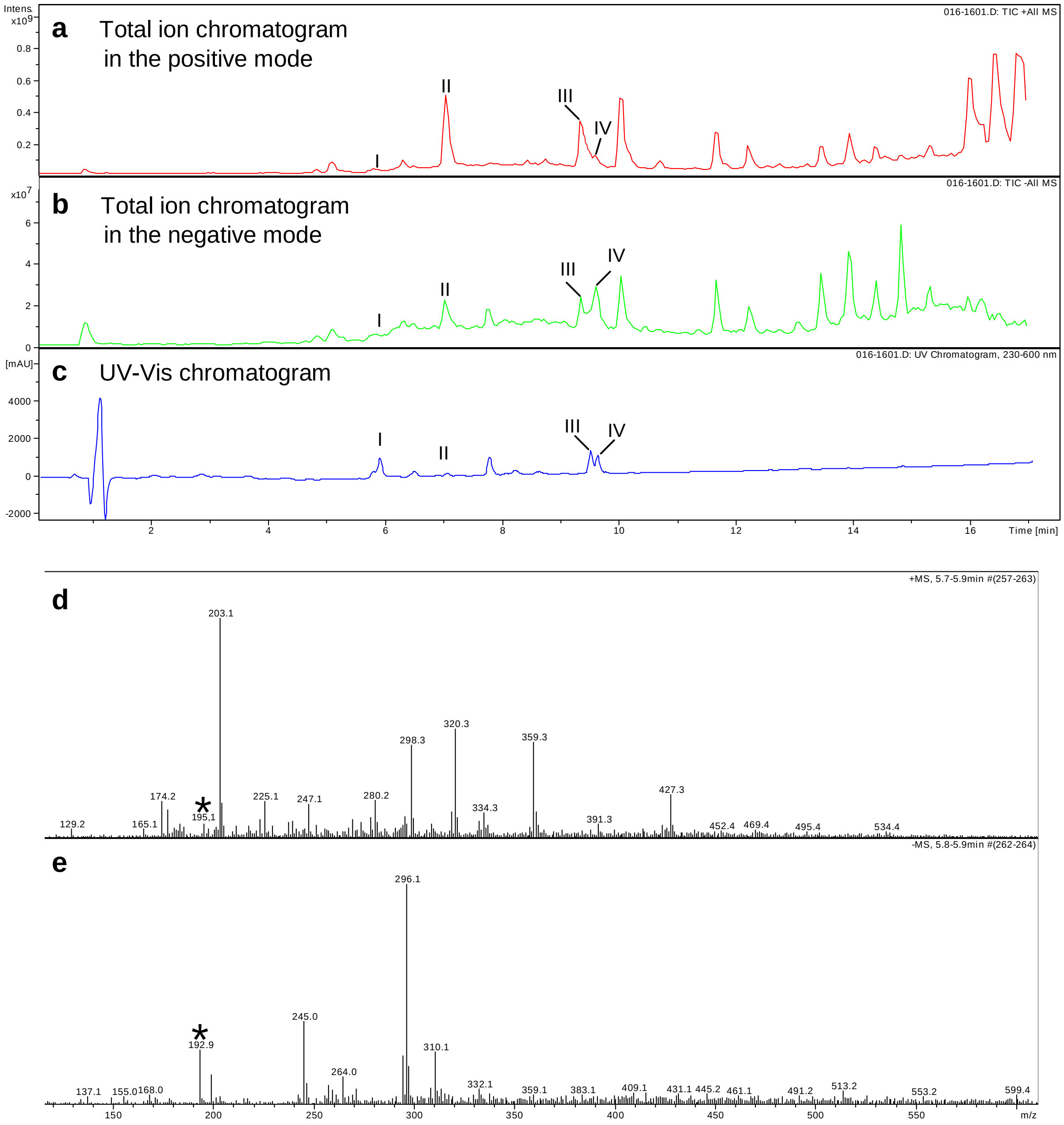

Additional File 2

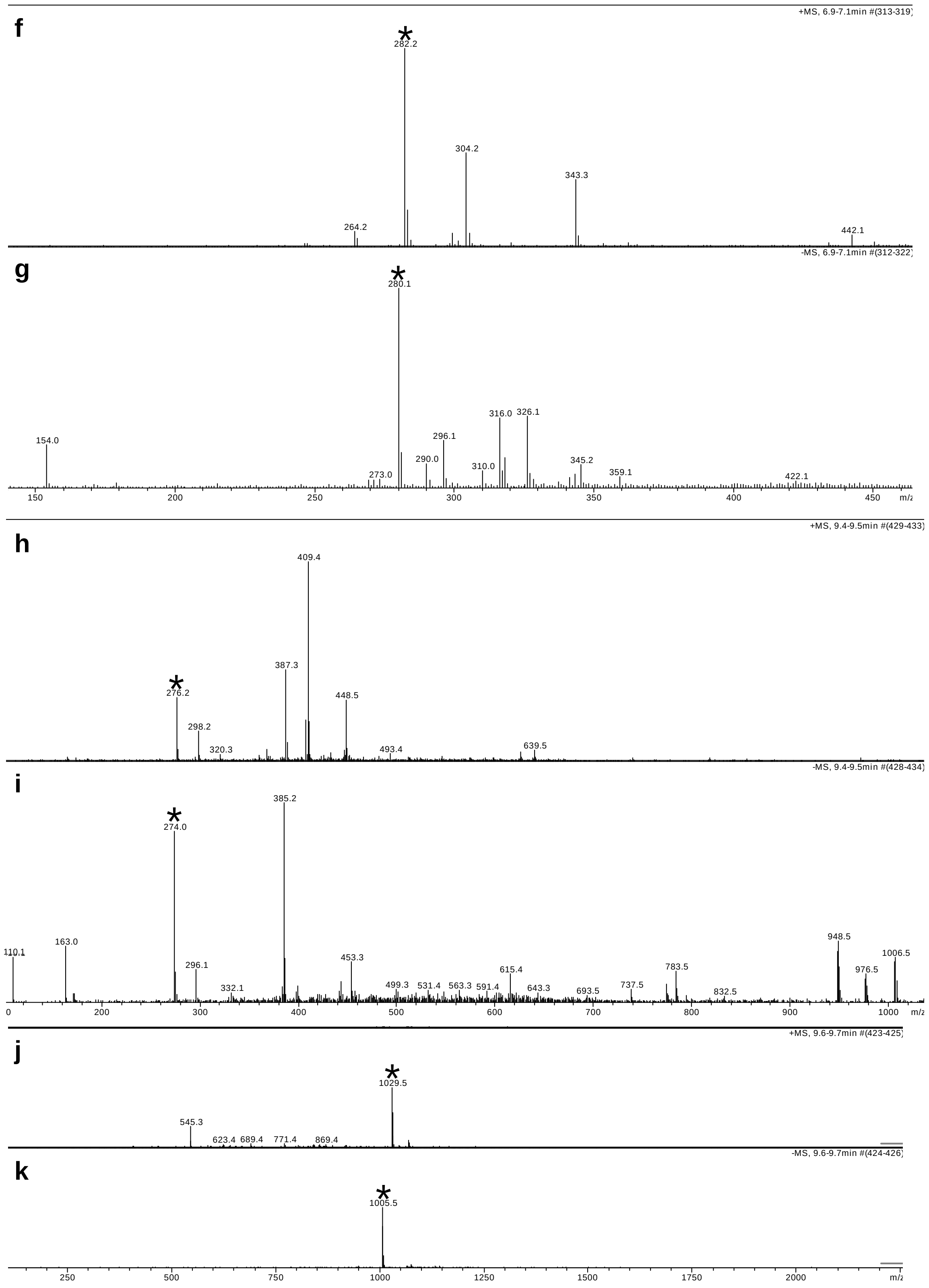

Additional File 2 Analysis of metabolites from *Streptomyces* sp. AcM11 Extracts were gained and analyzed as described in Methods. Total ion chromatograms at ESI-MS positive (a) and negative (b) modes, and UV-Vis spectrum at 230-600 nm (c) of organic extracts of *Streptomyces* sp. AcM11 suspension culture. The peaks I, II, III and IV are marked. The averaged masses of the ions within peaks I, II III, and IV are presented in ESI-MS positive (d, f, h, j) and negative (e, g, i, k) modes. The by MS and by comparisons to reference substance identified compounds are indicated by asterisks. Peak I was identified as ferulic acid (MW = 194.06), peak II as cycloheximide (MW = 281.16), peak III as actiphenol (MW = 275.12) , and peak IV as a derivative of Acta 2930-B1 (m/z = 1030.5 at [MS+H]+ and m/z = 1006.5 at [MS-H]-).
