# Supplementary material for: Production of fungal and bacterial growth modulating secondary metabolites is widespread among mycorrhiza-associated streptomycetes
Source: BMC Microbiol. 2012 Aug 2;12:164. doi: 10.1186/1471-2180-12-164 (PMC3487804; doi:10.1186/1471-2180-12-164)
Supplement: Additional file 3 — Streptomyces sp. AcM11 produces a derivative of Acta 2930-B1 Comparisons between the chromatogram and the averaged masses of the ions from Acta 2930-B1 pure substance and from peak IV of Streptomyces AcM11 extract, prepared as described in Methods. (a) The chromatogram of Acta 2930-B1 pure substance (blue) and the Streptomyces AcM11 extract (red). Average masses of Acta 2930-B1 pure substance and the Streptomyces AcM11 extract are in ESI-MS positive (b, d) and negative (c, e) modes. Note that the dominant masses in peak IV deviate one m/z unit from the respective values of the Acta 2930-B1 pure substance. [file 1471-2180-12-164-S3.pdf]

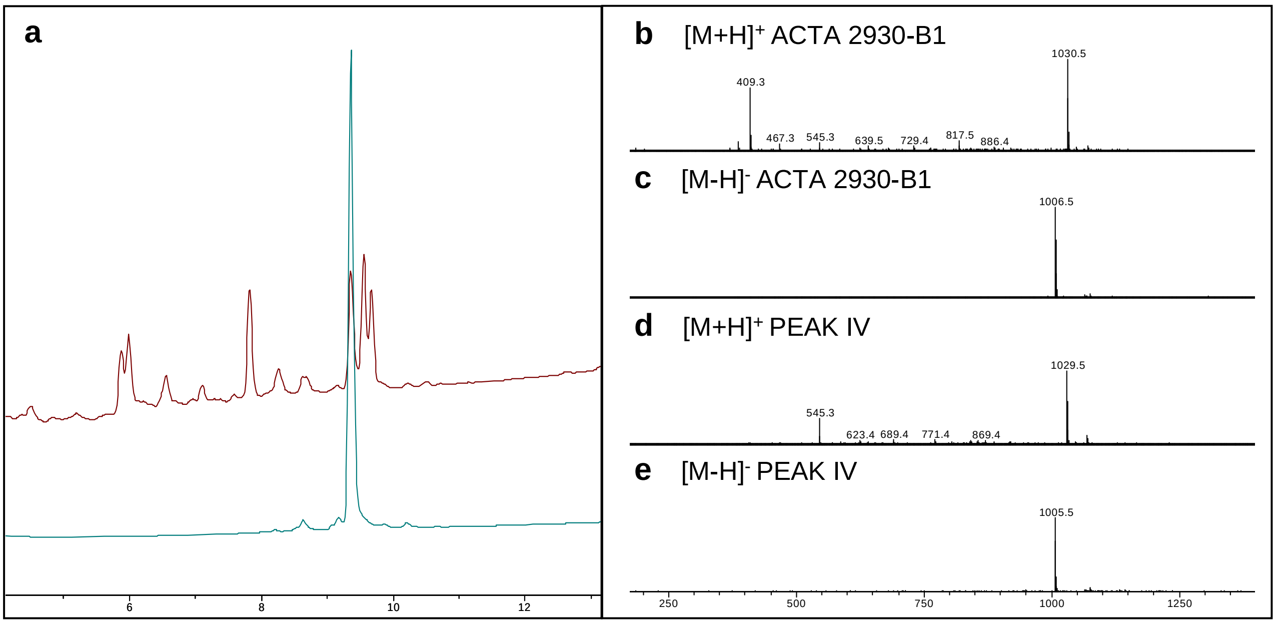

Additional File 3 *Streptomyces* sp. AcM11 produces a derivative of Acta 2930-B1

Comparisons between the chromatogram and the averaged masses of the ions from Acta 2930-B1 pure substance and from peak IV of *Streptomyces* AcM11 extract, prepared as described in Methods. (a) The chromatogram of Acta 2930-B1 pure substance (blue) and the *Streptomyces* AcM11 extract (red). Average masses of Acta 2930-B1 pure substance and the *Streptomyces* AcM11 extract are in ESI-MS positive (b, d) and negative (c, e) modes. Note that the dominant masses in peak IV deviate one m/z unit from the respective values of the Acta 2930-B1 pure substance.
