# Supplementary material for: Production of fungal and bacterial growth modulating secondary metabolites is widespread among mycorrhiza-associated streptomycetes
Source: BMC Microbiol. 2012 Aug 2;12:164. doi: 10.1186/1471-2180-12-164 (PMC3487804; doi:10.1186/1471-2180-12-164)
Supplement: Additional file 4 — Heterobasidion abietinum is more sensitive to the cycloheximide producer, Streptomyces AcM11, and to cycloheximide than H. annosum. Antifungal influence of AcM11 and cycloheximide was tested in a Petri dish bioassay test against H. abietinum 331 and H. annosum 005. (a, d) Influence of AcM11 on the growth of the fungus. AcM11 was applied on agar medium and the fungus was inoculated. The front of the fungal colony was circled by pencil. (b, e) Influence of cycloheximide on fungal growth. Methanol or in methanol dissolved cycloheximide was applied by filter paper on the top of the agar medium. Note that H. abietinum growth under the influence of 4 nmol cycloheximide is comparable to H. annosum growth with 50 nmol cycloheximide. The front of the fungal colony was circled by pencil. (c, f) Influence of cycloheximide on fungal growth on fungal growth. Extension of fungal mycelium was measured after one week of growth on cycloheximide containing medium (n = 9). Cycloheximide concentration range in the bioassay is based on the observed production level in the AcM11 suspension culture, which was 10.2 nmol x ml-1. Note the lower levels of cycloheximide applications to H. abietinum than to H. annosum. [file 1471-2180-12-164-S4.doc]

**ADDITIONAL FILE 4**

**Additional File 4 *Heterobasidion abietinum* is more sensitive to the cycloheximide producer, *Streptomyces* AcM11, and to cycloheximide than *H. annosum*.** Antifungal influence of AcM11 and cycloheximide was tested in a Petri dish bioassay test against *H. abietinum* 331 and *H. annosum* 005. (a, d) Influence of AcM11 on the growth of the fungus. AcM11 was applied on agar medium and the fungus was inoculated. The front of the fungal colony was circled by pencil. (b, e) Influence of cycloheximide on fungal growth. Methanol or in methanol dissolved cycloheximide was applied by filter paper on the top of the agar medium. Note that *H. abietinum* growth under the influence of 4 nmol cycloheximide is comparable to *H. annosum* growth with 50 nmol cycloheximide. The front of the fungal colony was circled by pencil. (c, f) Influence of cycloheximide on fungal growth on fungal growth. Extension of fungal mycelium was measured after one week of growth on cycloheximide containing medium (n = 9). Cycloheximide concentration range in the bioassay is based on the observed production level in the AcM11 suspension culture, which was 10.2 nmol x ml^-1^. Note the lower levels of cycloheximide applications to *H. abietinum* than to *H. annosum*.

**Methods**

**Application of antibiotic Acta 2930 B1, actiphenol, cycloheximide and siderophore ferulic acid on culture medium to test growth inhibition of *Heterobasidion* species**

The production levels of the antibiotics Acta 2930 B1, actiphenol, cycloheximide and the siderophore ferulic acid in *Streptomyces* sp. AcM11 suspension culture were quantified based on the comparison of their peak areas with those obtained by HPLC analysis of known amounts of the pure substance. The reference compounds were dissolved in methanol and applied on filter stripes (8 cm * 0.5 cm) at the concentration estimated as the AcM11 production level, as well as at higher concentrations. After the evaporation of methanol, the dry stripe was placed to the lower third of an agar-filled Petri dish. Agar plugs with fungal mycelium from the rim of two-week old culture were placed at the upper third of the Petri dish. Extension of fungal mycelium towards the stripe was measured after one week.
